# Supplementary material for: Transcriptomic Insights into Mechanisms of Early Seed Maturation in the Garden Pea (Pisum sativum L.)
Source: Cells. 2020 Mar 23;9(3):779. doi: 10.3390/cells9030779 (PMC7140803; doi:10.3390/cells9030779)

Zhongwan−6,25(4)

Global Summary

%DE = 0.25

genes with fdr < 0.2 = 155 (67 + / 88 −)

genes with fdr < 0.1 = 144 (63 + / 81 −)

genes with fdr < 0.05 = 137 (60 + / 77 −)

genes with fdr < 0.01 = 124 (52 + / 72 −)

genes in genesets = 0

<FC> = 0

<t−score> = 0

<p−value> = 0

<fdr> = 0.76

Portrait

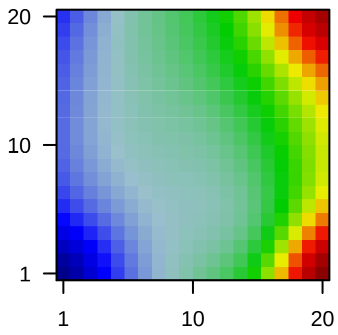

Top 100 DE genes

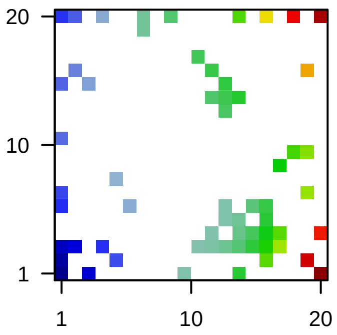

Global Genelist

Rank	ID	log(FC)	p−value	fdr	Metagene	Description
1	TRINITY_DN2	−0.17	2e−16	2e−15	14 x 3	
2	TRINITY_DN1	5.71	2e−16	2e−15	20 x 1	
3	TRINITY_DN1	3.01	2e−16	2e−15	20 x 20	
4	TRINITY_DN5	0.7	2e−16	2e−15	2 x 20	
5	TRINITY_DN5	0.23	2e−16	2e−15	13 x 14	
6	TRINITY_DN5	0.15	2e−16	2e−15	1 x 11	
7	TRINITY_DN2	2.73	2e−16	2e−15	20 x 1	
8	TRINITY_DN2	1.3	2e−16	2e−15	20 x 1	
9	TRINITY_DN3	−0.29	2e−16	2e−15	1 x 1	
10	TRINITY_DN3	0.14	2e−16	2e−15	19 x 10	
11	TRINITY_DN5	−0.15	2e−16	2e−15	14 x 4	
12	TRINITY_DN4	−0.45	2e−16	2e−15	15 x 3	
13	TRINITY_DN4	−1.13	2e−16	2e−15	1 x 1	
14	TRINITY_DN4	1.68	2e−16	2e−15	18 x 20	
15	TRINITY_DN1	−0.17	2e−16	2e−15	1 x 1	
16	TRINITY_DN3	0.24	2e−16	2e−15	20 x 1	
17	TRINITY_DN2	−0.17	2e−16	2e−15	1 x 2	
18	TRINITY_DN2	−0.26	2e−16	2e−15	1 x 1	
19	TRINITY_DN4	−0.57	2e−16	2e−15	1 x 1	
20	TRINITY_DN1	−0.21	2e−16	2e−15	16 x 5	

p−values

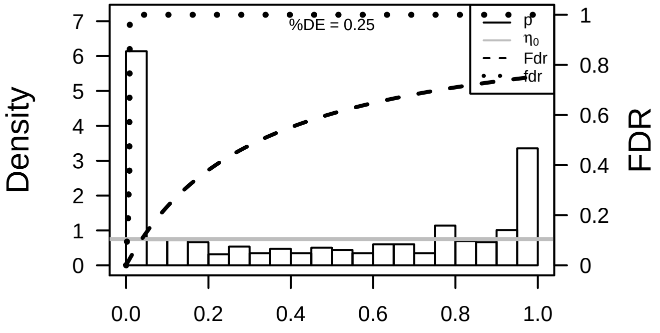

Supplement: Supplementary file 1 [file cells-09-00779-s001.zip › Supplementary materials/FileS3.zip!/FileS2/Hormones - Results/Summary Sheets - Samples/Zhongwan.6.25.4..pdf]
